# Effect of Solution Treatment on the Microstructure and Elevated Temperature Tensile Properties of Forged Rene 41 Superalloy

**DOI:** 10.3390/ma17246150

**Published:** 2024-12-16

**Authors:** Xianguang Zhang, Haoran Han, Yang Zhou, Jiajun Chen, Shouli Feng, Pingmei Tang, Dongping Xiao, Jianhui Fu, Jian Zhang

**Affiliations:** 1 School of Metallurgical and Ecological Engineering, University of Science and Technology Beijing, Beijing 100083, China; 2 Chengdu Advanced Metal Materials Industrial Technology Institute Co., Ltd., Chengdu 610300, China

**Keywords:** Rene 41 superalloy, solution treatment, microstructure, elevated mechanical properties

## Abstract

The effects of a solution treatment on the microstructure and elevated mechanical properties of the forged Rene 41 superalloy were investigated. The results indicate that the solution treatment temperature has a significant influence on the γ′ structure and mechanical properties. The sub-solvus solution treatment resulted in the co-existence of residual primary coarse γ′ precipitates and fine secondary γ′ precipitates, while the super-solvus solution treatments led to the complete dissolution of the primary γ′ precipitates and the precipitation of a nano-sized secondary spherical γ′ precipitate. The tensile strength increased and then decreased when the solution temperature increased from the sub-solvus to super-solvus solution treatments. In addition, the solution treatment time has a negligible influence on the γ′ and overall mechanical properties due to the complete dissolution of γ′ during the solution treatment at 1080 °C for 1 h. Moreover, the cooling rate following the solution treatment plays a significant role regarding the size and morphology of γ′ and the mechanical properties. The secondary γ′ changed gradually from spherical to concave cubic and octo-cubic and coarsened with the decrease in the cooling rate, resulting in an apparent decrease in strength and increase in ductility.

## 1. Introduction

The Rene 41 superalloy has been widely used in aeroengines [1,2,3]. The harsh service conditions require the alloy to have excellent elevated-temperature mechanical properties [4,5,6,7]. It was reported that the strength and plasticity depend on the size and morphology of the γ′ grains and carbides for the Rene 41 alloy, which are strongly influenced by the heat treatment [8,9,10,11,12], and the solution treatment is a key process for it. Therefore, it is crucial to investigate the solution treatment response of the Rene 41 alloy to help in tailoring the excellent high-temperature mechanical properties.

Previously, researchers studying the solution treatment of the Rene 41 superalloy were focused on cold-rolled or additively manufactured Rene 41 alloys [13,14,15]. Li et al. [13] conducted a study on the heat treatment behavior of a cold-drawn Rene 41 alloy and found that the high-temperature solution and high-temperature aging treatments produced coarse grains and secondary γ′ and a larger amount of grain boundary carbide, leading to relatively low tensile strength. Atabay [14] studied the solution treatment response of an additively manufactured Rene 41 alloy by laser powder bed fusion and found that the sub-solvus solution treatment led to a bimodal distribution of γ′ and a discrete distribution of the grain boundary carbides, while, during the super-solvus solution treatment, the grains were transformed from columnar to equiaxed ones and the formation of film-like grain boundary carbides occurred. It was also reported that the 4 h 1065 °C- treatment, which is slightly above the solution temperature, could not completely dissolve the primary γ′ and carbides for the additively manufactured Rene 41 alloy [15].

The above studies provide certain guidance for understanding the solution treatment of the forged Rene 41 alloy. However, the initial structure and grain size are significantly different from those of the forged alloy, and the research on the solution treatment of the forged Rene 41 alloy is scarce. The Rene 41 alloy is a typical nickel-based superalloy containing carbides. Previously, researchers studied the solution treatment of superalloys containing carbides. It was found that the grain growth and carbide distribution depend on the solution temperature. For example, it was reported that the grain growth of the GH4738 alloy was sluggish during the sub-solvus solution treatment (lower than 1040 °C), while it became obvious during the super-solvus solution treatment (higher than 1040 °C) [16]. Hu [17] found that increasing the solution temperature and prolonging the holding time of GH4738 caused the grains to grow appropriately and then proposed that the grains can be controlled by adjusting the solution temperature and holding time.

On the other hand, it was acknowledged that both the grain and carbide sizes increased with the increase in the solution treatment temperature [18,19]. For example, it was found that the grain structure and grain boundary carbides of GH2150A are fine and uniform at solution temperatures between 1020 °C and 1040 °C (super-solvus solution temperature), and the grain size and grain boundary carbides increase as the solution temperature increases. Zhao [20] determined that the alloy grain size depends on the maximum treatment temperature during the heat treatment of the GH2150A alloy. In addition, Rong [21] studied the effect of the cooling rate following the solution treatment on the microstructure of the GH4738 alloy and it was found that the cooling rate had a negligible influence on the grain size, while the γ′ content and size gradually decreased with the increase in the cooling rate.

The above studies demonstrate that the solution treatment temperature, time, and cooling rate following the solution treatment have important influences on the γ′ and grain size for the cold-rolled manufactured Rene 41 alloy or other carbide-containing wrought superalloys. However, the influence of the solution treatment on the γ′ and elevated mechanical properties of the forged Rene 41 alloy remains unclear. Therefore, this study aimed to study the effects of the solution treatment on the microstructure and elevated mechanical properties of the forged Rene 41 alloy.

## 2. Experimental Materials and Methods

**Figure 1:**
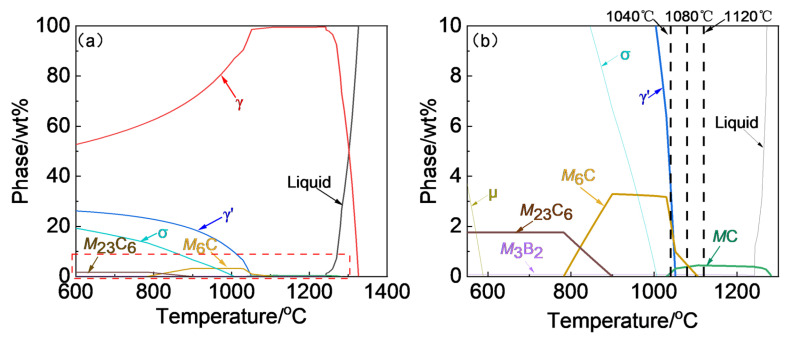
(**a**) Overall and (**b**) partially enlarged phase diagram of Rene 41 superalloy.

**Figure 2:**
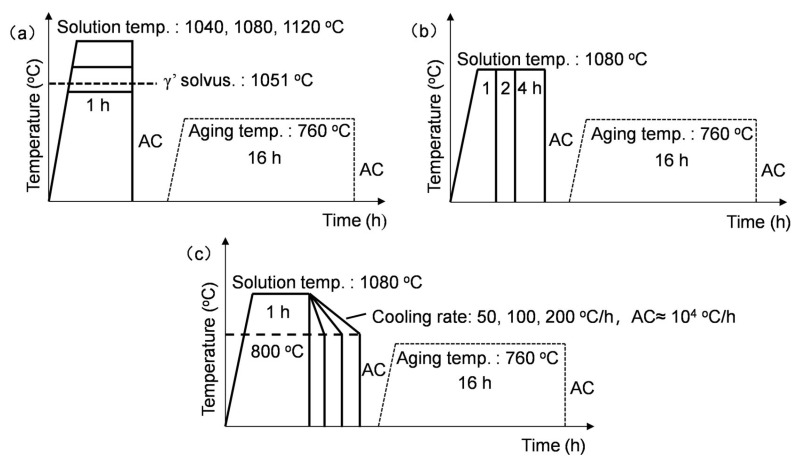
Thermal histories for the heat treatments with varying (**a**) solution temperatures, (**b**) solution treatment times, and (**c**) cooling rates following the solution treatment for the forged Rene 41 superalloy.

The chemical composition of the as-forged Rene 41 superalloy used in this work is shown in Table 1, and the radial forging was carried out after the homogenization and clogging treatments. The equilibrium phase diagram calculated by JMatPro (v.9.0) is shown in Figure 1, and the equilibrium dissolution temperature of γ′ was predicted to be 1051.7 °C. As mentioned above, the main purpose of the solution treatment is to dissolve the coarse primary γ′. Therefore, three solution treatment temperatures of 1040 °C, 1080 °C, and 1120 °C, slightly below or above the γ′ dissolution temperature, were selected to study the effects of solution temperature, as shown in Figure 2a. The thermal histories for investigating the effects of solution time and cooling rate following the solution treatment are shown in Figure 2b,c. The solution-treated alloys were cooled to 800 °C at varying cooling rates and then air-cooled to room temperature. The aging conditions are the same for all the cases.

The heat treatments were carried out in a high-temperature box-type resistance furnace. The samples cut from the heat-treated samples with the size of 10 mm × 5 mm × 2 mm were used for the microstructure characterization. Chemical etching (3 g CuSO_4_+40 mL HCl + 3 mL H_2_SO_4_, 30–40 s) after mechanical polishing and grinding were carried out to reveal the grain boundaries and γ′. Optical microscope (OM, CX40M, SUNNY GROUP, Yuyao, China) and scanning electron microscope (SEM, JSM 7200F, JEOL, Tokyo, Japan) equipped with an X-ray energy spectrometer (EDS, NS7, JEOL, Tokyo, Japan) were used for the microstructure characterization. Some samples were characterized by electron backscattered diffraction (EBSD, MIRA3 LMH, TESCAN, Brno, Czech Republic) and transmission electron microscopy (TEM, FEI Tecnai F20, Hillsboro, OR, America) equipped with EDS. To remove the surface damage after mechanical polishing, electrolytic polishing (20 mL HCl + 80 mL C_2_H_5_OH, 20 V, 10 s) was conducted prior to EBSD analyses. The thin-film TEM samples were prepared by twin-jet polishing (Struers Tenupol-5, 10% HClO_4_ + 90% C_2_H_5_OH, 20 V, −25 °C). The high-temperature tensile tests were performed at 760 °C at a rate of 1 mm/min, with a holding time of 5 min before the tensile test.

## 3. Results and Discussion

### 3.1. Initial Structure Characterization

**Figure 3:**
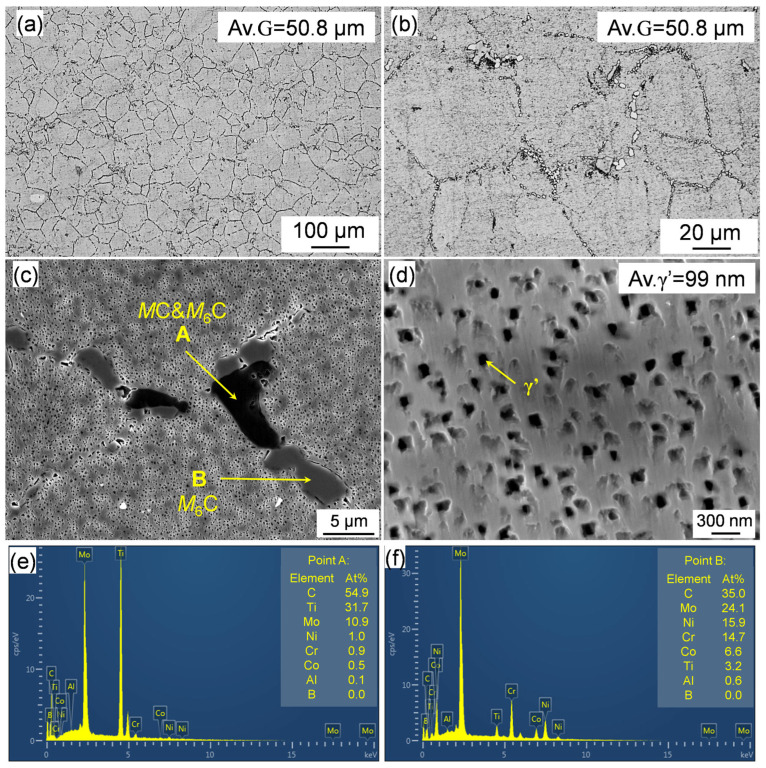
(**a**,**b**) OM and (**c**,**d**) SEM images showing the carbides and γ′ of the as-forged Rene 41 alloy; (**e**,**f**) EDS point analyses of the areas marked A and B in (**c**).

The microstructure of the as-forged Rene 41 alloy before the heat treatment is shown in Figure 3. It can be seen from the OM images (Figure 3a,b) that the grain structure is relatively uniform, and the average grain size is approximately 50.8 μm. According to Figure 3b, there are many chain-like and large-size granular precipitates distributed at the grain boundaries. The high-magnification SEM image and corresponding EDS-analyzed results are shown in Figure 3c,f. The EDS measurements confirmed that the dark precipitates in the SEM image are Ti- and Mo-enriched and the gray ones are Mo- and Cr-enriched. This indicates that the dark and gray precipitates are MC+M_6_C and M_6_C carbides, respectively, according to the previous work [22]. In addition, relatively coarse γ′ precipitates are clearly observed, and the size of the γ′ was measured to be 99 nm. It is known that the coarse carbides and γ′ determine the mechanical properties [23]. Therefore, a solution treatment is necessary to be carried out to dissolve the coarse primary γ′ and excessive carbides and re-precipitate fine secondary γ′ to improve the mechanical properties.

### 3.2. Influences of Solution Treatment Temperature and Time

**Figure 4:**
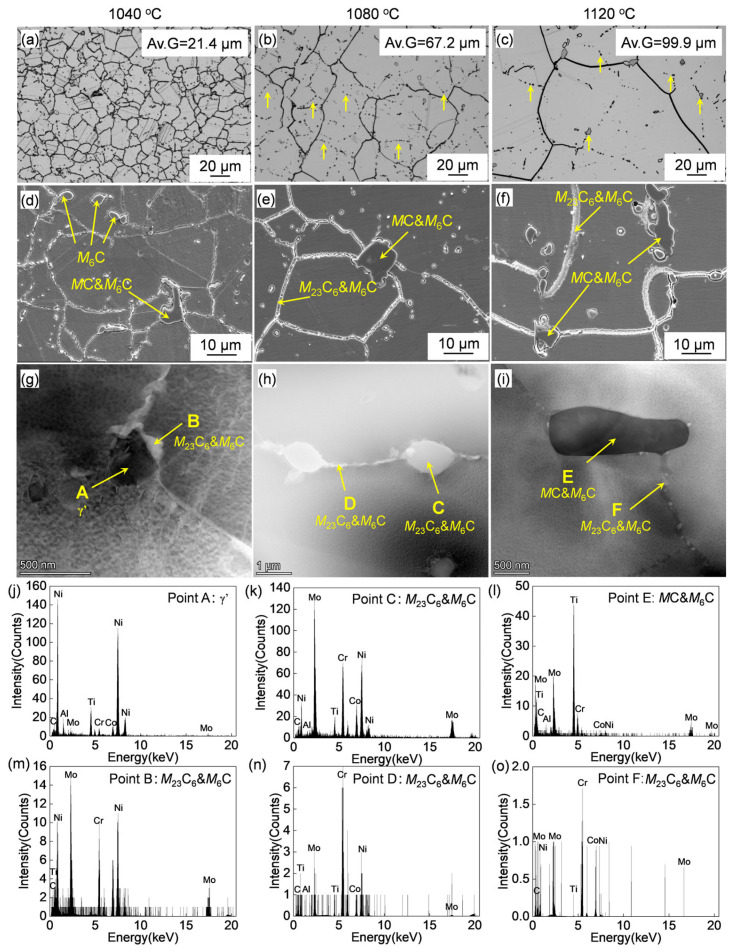
(**a**–**c**) OM and (**d**–**f**) SEM images of the forged Rene 41 alloy after solution treated at varying temperatures; (**g**–**i**) HAADF images of the precipitates and (**j**–**o**) corresponding EDS point analysis results.

The OM and SEM images of the forged alloy after solution treatments at varying temperatures are shown in Figure 4. As the solution temperature increases, the grain size gradually increases. At 1040 °C, the sub-solvus solution treatment condition, the average grain size was measured to be 21.4 μm, which is smaller than that of the as-forged initial structure. This is probably due to the static recrystallization that occurs under the high-temperature solution treatment, attributed to the residual strain remaining in the as-forged ingot [24]. At the super-solvus solution temperature of 1080 °C, the average grain size is 67.2 μm, which is close to that of the initial structure. As the solution treatment was performed at 1120 °C, the average grain size was 99.9 μm. The grain growth that occurred with the increasing solution treatment temperature decreased the total grain boundary area and energy [25].

In addition, some black precipitates were observed in the OM images. The SEM images show that these precipitates are distributed at the grain boundaries and are intragranular. The HADDF images and STEM-EDS-analyzed results are shown in Figure 4g,i. The gray precipitates (as marked by B,C,D) under the HADDF are Mo- and Cr-enriched, while the black precipitates (as marked by E) are Ti- and Mo-enriched, which indicates that they are the M_6_C+ M_23_C_6_ and MC+ M_6_C carbides, respectively, according to the previous research [22]. On the other hand, the dark precipitate, as marked by A in Figure 4g, is the coarse primary γ′. The equilibrium dissolution temperature of the MC carbide is 1283 °C according to the phase diagram in Figure 1; therefore, the MC carbide cannot be completely dissolved regardless of the homogenization or forging treatments, and the presence of the MC carbide could be observed after the heat treatment. On the other hand, according to Theska’s research [26], the M_23_C_6_ on the grain boundaries may be formed due to the segregation of the grain boundary elements during the cooling process following the solution and subsequent aging treatments.

The grain boundary carbides play dual roles regarding the mechanical properties of the alloy: beneficial for grain boundary pinning but potentially detrimental for ductility. The grain boundary carbides play a beneficial role in reducing the grain growth or blocking dislocation motion, which is helpful for grain refinement and increasing the grain boundary strength [27]. On the other hand, the grain boundary carbides have detrimental effects as stress concentrators, reducing the ductility and making the alloy more susceptible to cracking [28].

**Figure 5:**
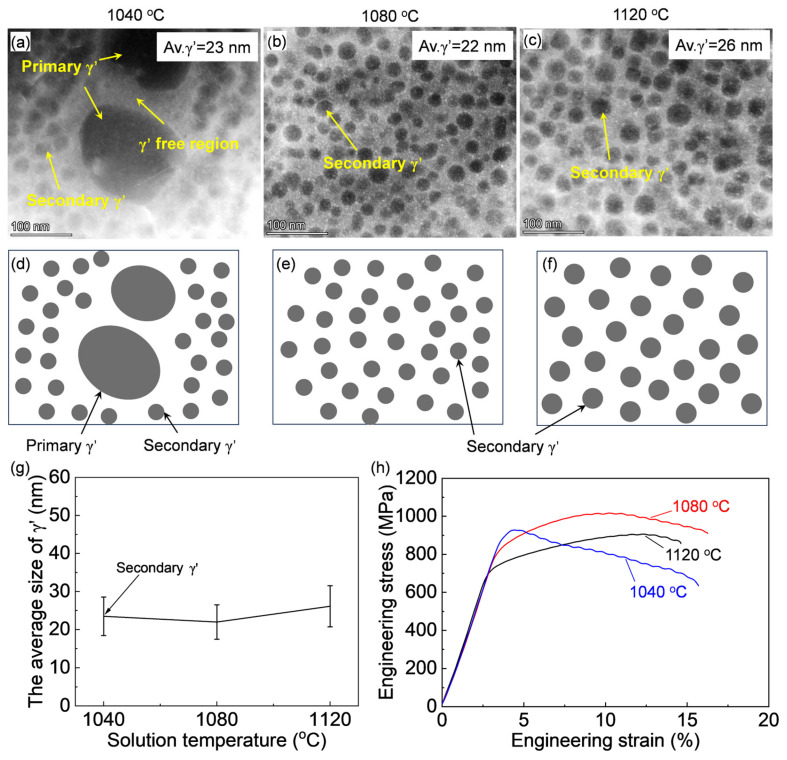
(**a**–**c**) HAADF images of γ′ after solution treated at varying temperatures and the (**d**–**f**) schematic illustrations of the γ′ morphology and size. (**g**) Quantitatively analyzed changes in γ′ size against solution treatment temperatures. (**h**) Stress–strain curves of the alloy at 760 °C after different solution treatments at varying temperatures.

The γ′ structures were characterized by TEM, as shown in Figure 5. According to Figure 1, the equilibrium dissolution temperature of γ′ is approximately 1051.7 °C. After the sub-solvus solution treatment at 1040 °C (Figure 5a), nano-sized secondary γ′ precipitates with an average diameter of 23 nm were newly precipitated; meanwhile, the primary γ′ precipitates could not be completely dissolved, and some of the primary γ′ precipitates remained. It is worth noting that there is a γ′-free region surrounded by the coarse primary γ′. In the case of the super-solvus solution treatments (Figure 5b,c), the nano-sized secondary γ′ is uniform without the observation of the coarse primary γ′. The schematic illustrations of the changes in the γ′ morphologies and quantitatively analyzed γ′ size and volume fraction (ƒ) under varying solution treatment temperatures are shown in Figure 5d–g and Table 2. Spherical secondary γ′ precipitates were formed for the three cases, and the size of γ′ increased slightly with the increase in the solution treatment temperature but with a close fraction. This is well reflected in the changes in the mechanical properties.

Figure 5h shows the high-temperature tensile curves of the alloys after varying solution treatments. The alloy solution treated at 1080 °C exhibited the highest tensile strength of 1017.1 MPa, while the tensile strength of the solutions treated at 1040 °C and 1120 °C are 927.9 MPa and 906.9 MPa, respectively, sharing similar total elongation values.

According to the strengthening mechanisms, the flow stress (*σ*) during the tensile test is contributed by various strengthening mechanisms, including grain boundary strengthening (*σ_Gb_*), solution strengthening of γ matrix (*σ_Ss_*), precipitation strengthening of γ′ precipitates (*σ_Pre_*), and dislocation strengthening (*σ_Dis_*), and it can be summarized as in the following formula [29,30]:

(1) $$\begin{array}{c} \sigma =\sigma_{Gb}+\sigma_{Ss}+\sigma_{Pre}+\sigma_{Dis} \end{array}$$

The same alloy and similar heat treatments were used in this work; therefore, the solution strengthening of γ matrix (*σ_Ss_*) and dislocation strengthening (*σ_Dis_*) were considered to be the same. The differences in strength after varying solution treatments should be attributed to differences in the size of the grain and γ′.

Some studies indicated [31,32,33] that the γ′ phase size affected the strengthening mechanisms. When the γ′ phase size is small (68.5 nm, for example) [34], dislocations pass through the γ′ particles via a cutting mechanism, and the dislocations cut the γ′ particle, causing the γ′ phase and the matrix to be deformed simultaneously. The shear stress via the cutting mechanism is shown in Equation (2) as follows [35]:

(2) σPre=1.273Tbr×ƒ121.137−ƒ12

where *b* indicates material constants representing the Burgers vector’s magnitude. *T* is the dislocation line tension equaling *Gb*^2^/2 in numerical terms. *r* and ƒ represent the average size and volume fraction of the γ′ phase.

In addition, the grain boundary strengthening can be described as [36,37,38]

(3) σGb=k×d−12

where *k* is an experimentally estimated constant related to the material properties and *d* is the average grain size.

According to Equation (2), the precipitation strengthening is inversely proportional to the γ′ size, *r*, under a similar faction, and the grain boundary strengthening is inversely proportional to the grain size, *d*. During the sub-solvus solution treatment at 1040 °C, the large-sized primary γ′ precipitates could not be completely dissolved, and some of the coarse primary γ′ precipitates remained (Figure 5a), and these large-sized γ′ precipitates reduced the precipitation strengthening according to Equation (2). Although the solution treatment at 1040 °C resulted in a finer grain size, lower strength was obtained compared with the super-solvus solution treatment at 1080 °C. This indicates that precipitation strengthening is the main strengthening mechanism for the Rene 41 alloy. During the solution treatment at 1120 °C, both the γ′ and grains were slightly coarsened, resulting in a reduction in precipitation and grain boundary strengthening compared with the 1080 °C condition. Therefore, the solution treatment at 1080 °C achieved the optimal tensile strength among them due to the fine γ′ and grain size.

**Figure 6:**
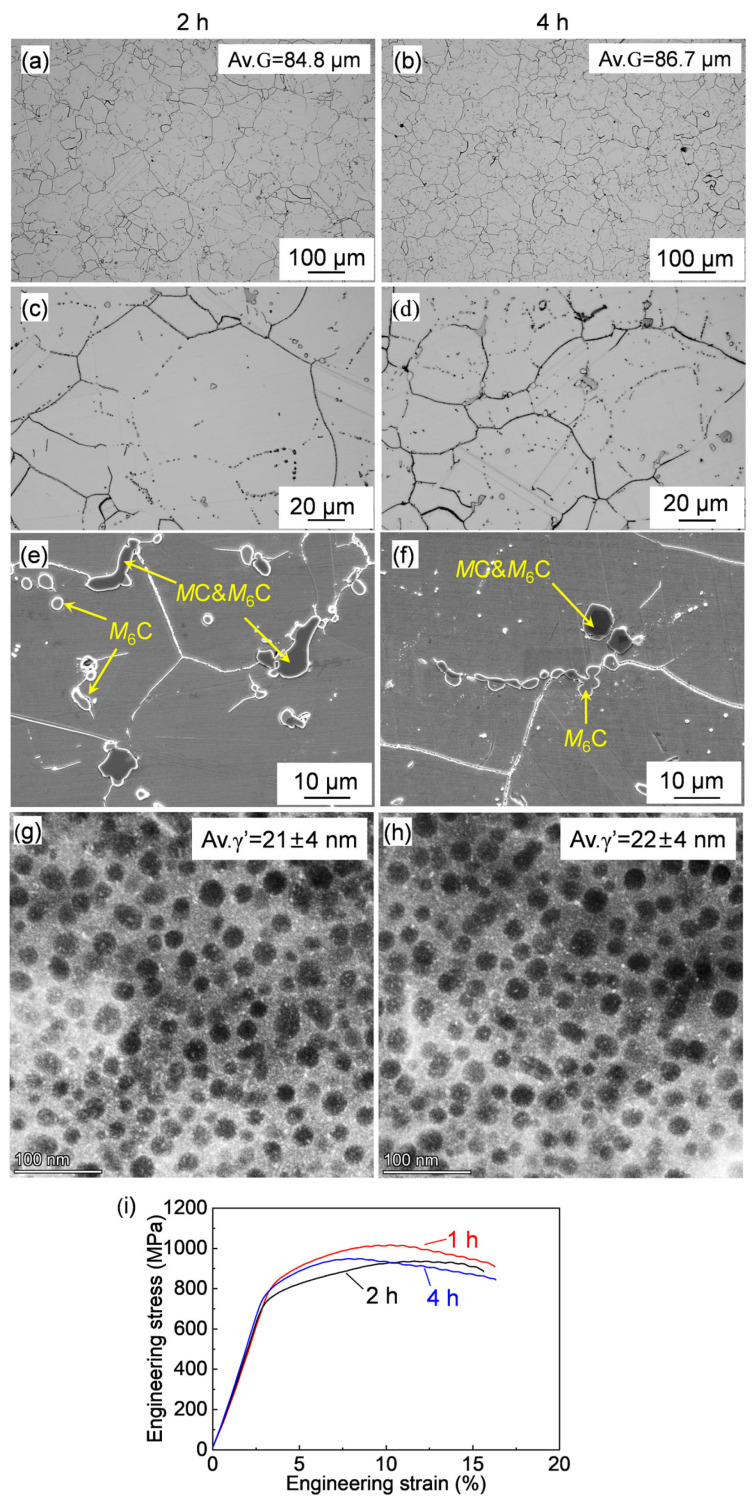
(**a**–**d**) OM and (**e**,**f**) SEM images of the forged Rene 41 alloy after solution treated at 1080 °C for varying periods. (**g**,**h**) HAADF images showing the γ′ and (**i**) stress–strain curves of the alloy at 760 °C.

The OM images of the samples after the solution treatment at 1080 °C for varying periods are displayed in Figure 6a–d. The extension of the solution treatment time caused a certain grain coarsening. The average grain size is around 67.2 μm after 1 h of solution treatment (Figure 4b), and the average grain sizes are 84.8 μm and 86.7 μm regarding the 2 h and 4 h solution treatments, respectively. The grain growth behavior is dependent on the solution temperature and time. Grain growth is more sensitive to temperature than time [39], and the grain size at a certain temperature could be reached at a steady state, viz. reaching a constant grain size against time. This is reflected in the grain growth during the solution time increasing from 1 h to 2 h, while, with a further increase in the solution time from 2 h to 4 h, the grain size reached a steady state and displayed little change. The negligible difference in the average grain sizes between the solution times of 2 and 4 results from normal fluctuation.

On the other hand, the extension of the solution time had a negligible influence on the γ′ precipitation, as shown in Figure 6g,h. Figure 6i shows the strain and stress curves of the alloys measured at 760 °C after the solution was treated at 1080 °C for varying periods. Evidently, the solution time had a weak influence on the tensile properties of the forged Rene 41 superalloy. According to Equations (1)–(3), the solid solution, dislocation, and precipitation strengthening are similar. The grains were slightly coarsened with the increase in the solution treatment period, resulting in a slight reduction in the grain boundary strengthening and therefore a slight decrease in the tensile strength.

**Figure 7:**
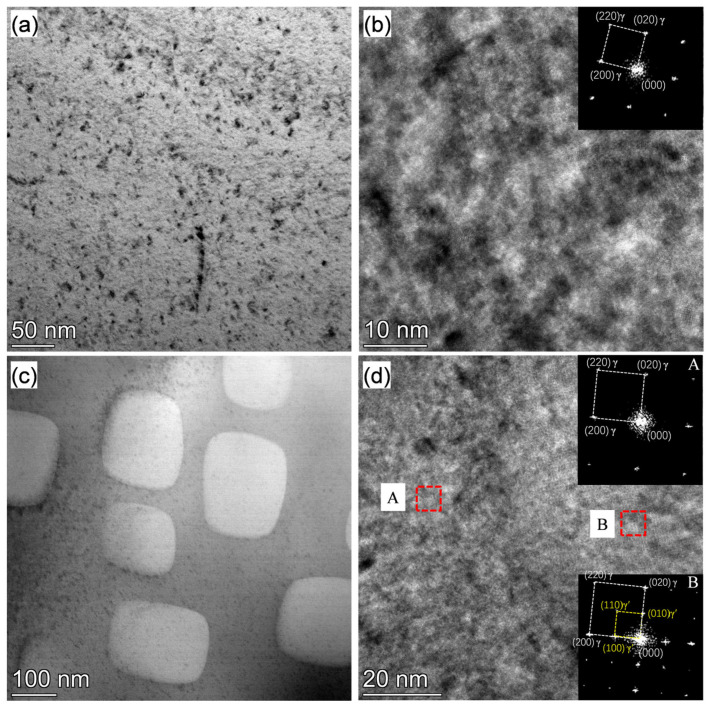
TEM bright field images of the (**a**) water-quenched after solution treated at 1080 °C for 1 h and (**c**) as-forged Rene 41 alloy, and (**b**,**d**) high-resolution TEM and its FFT-analyzed results.

In order to confirm the complete dissolution of γ′ after the solution treatment at 1080 °C for 1 h, the solution-treated samples were immediately quenched with water just after the solution treatment, and the TEM observation was applied to verify the existence of γ′. The typical TEM bright field images are shown in Figure 7. No γ′ precipitates could be observed after the solution treatment at 1080 °C for 1 h according to the high-resolution TEM and FFT analyses in which only the FCC matrix was detected, in contrast to the clear observation of the cubic γ′ in the as-forged state. Therefore, it can be concluded that the primary γ′ phase was completely dissolved after the 1 h 1080 °C solution treatment.

**Figure 8:**
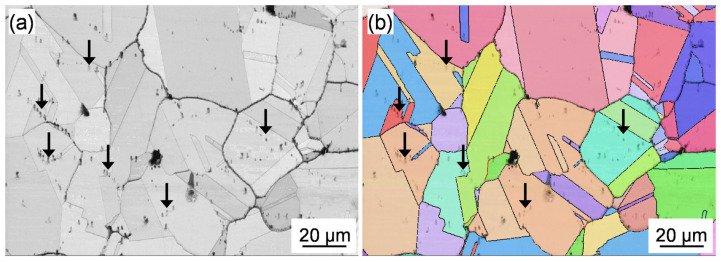
(**a**) IQ and (**b**) IPF maps showing the distribution of the ‘chain-like’ carbides in the heat-treated forged Rene 41 alloy.

According to Figure 4b,c, there are some ‘chain-like’ carbides distributed within the grain, which indicate that the carbides were precipitated at some specific grain boundaries, as shown by the arrows in Figure 4b,c. To verify the existence of specific grain boundaries, EBSD analyses were carried out. The typical image quality (IQ) and inverse pole figure (IPF) maps of the heat-treated Rene 41 superalloy analyzed by EBSD are shown in Figure 8. The trace of the ‘chain-like’ carbides is clearly observed in the IQ map, as indicated by the black arrows. The corresponding IPF map shows that there are no specific grain boundaries, and the ‘chain-like’ carbides are just distributed intragranularly, indicating that the ‘chain-like’ carbides correspond to the undissolved carbides of the original forged ingot, which remained at the prior grain boundaries. According to the equilibrium phase diagram (Figure 1), the complete dissolution temperature of the MC carbides is about 1283 °C, which is in the liquid phase. Therefore, the solution treatments performed at 1040 °C, 1080 °C, and 1120 °C cannot completely dissolve the MC carbide, and some of the MC carbides in the as-forged state remained even after the solution treatment (Figure 8).

### 3.3. Influences of Cooling Rate After Solution Treatment

**Figure 9:**
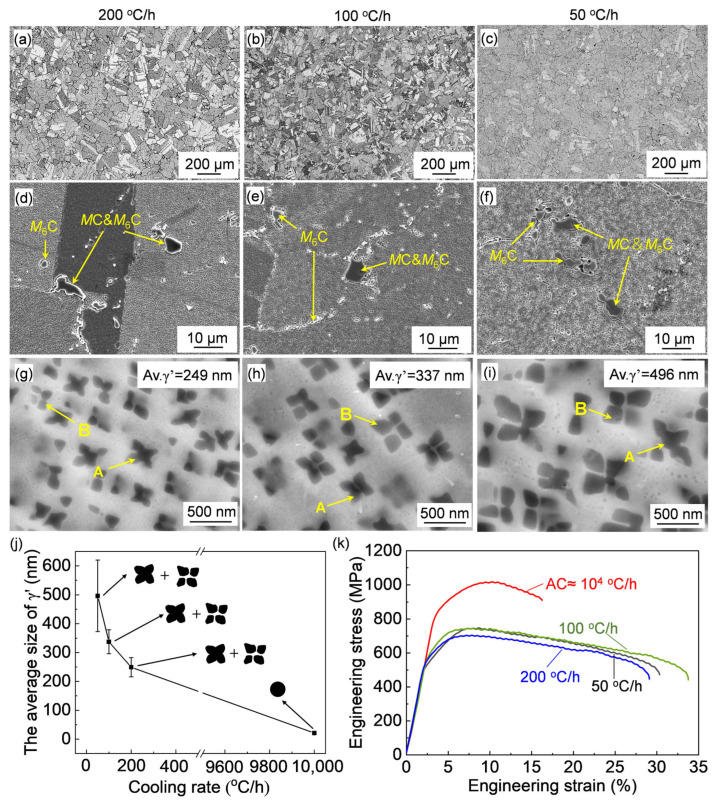
(**a**–**c**) OM and (**d**–**f**) SEM images of the forged Rene 41 alloy after solution treated at 1080 °C and cooled at varying cooling rates following solution treatment. (**g**–**i**) HAADF images showing the changes in γ′ morphologies and (**j**) the quantitatively analyzed changes in γ′ size against cooling rates. (**k**) The stress–strain curves of the alloy at 760 °C after solution treated and cooled at varying cooling rates following solution treatment.

The OM and SEM images of the alloys cooled at different cooling rates following the solution treatment are shown in Figure 9. Similar grain structures were obtained after solution treatments at varying cooling rates, indicating that the cooling rate has a negligible influence on the grain size. The distribution of the carbides after cooling is similar as well; therefore, the effect of the cooling rate on the carbide distribution is negligible.

Although the cooling rate has weak a influence on the grains and carbides, it has a significant influence on the size and morphology of γ′. As the cooling rate decreases, the γ′ gradually coarsens and changes in morphology, as indicated in Figure 9g–i. At the cooling rate of 100 °C/h, the γ′ precipitates were formed in the concave cubic (arrow A in Figure 9g) and octo-cubic (arrow B in Figure 9g) morphologies, with average sizes of 249 nm. Therefore, the morphologies of γ′ changed from spherical to cubic when the cooling rate decreased from 10^4^ °C/h (air-cooling, Figure 5b) to 100 °C/h. With the further decrease in the cooling rate, the ratio of octo-cubic γ′ increased. The quantitatively analyzed changes in the γ′ size and volume fraction against the cooling rate are displayed in Figure 9j and Table 3, and the corresponding morphology of γ′ is schematically drawn in Figure 9j as well. The γ′ gradually coarsened and slightly decreased in faction with the decrease in the cooling rate.

The high-temperature tensile curves of the alloys under different cooling rates are shown in Figure 9k. The slow cooling resulted in obvious decreases in the yield and tensile strength in comparison with the air-cooling case, while a dramatic increase in the total elongation occurred, nearly twice that of the air-cooled case. Specifically, the tensile strength of the slowly cooling sample at 50 °C/h is 747.4 MPa and the elongation is 30.3%. The tensile strength values of the slow-cooling samples at 100 °C/h and 200 °C/h are 744.4 MPa and 704.0 MPa, respectively, and the elongation values are, respectively, 33.8% and 29.0%.

The resultant similar grain sizes under different cooling rates and the same alloy and similar heat-treatment cycles were used; therefore, the grain boundary strengthening, solid-solution, and dislocation strengthening are close for the cases of varying cooling rates. Hence, the differences in the elevated mechanical properties should be attributed to the differences in γ′ according to the strengthening model in Equation (1). The slow cooling resulted in coarse γ′ precipitates with a size of hundreds of nm. As mentioned above, as for the tens of nano-sized γ′ precipitates, dislocations pass through the γ′ particles by a cutting mechanism [34]. When the γ′ gradually coarsens, the dislocations pass through the second-phase particles via a by-pass mechanism (68.5 nm) [34], forming Orowan loops that interact with the γ′ phase, causing the γ′ phase to deform, viz. the Orowan mechanism. The Orowan looping (*τ_orowan_*) is described as follows [40]:

(4) τorowan=3Gb2r×2π3ƒ−12

where *r* and ƒ represent the average size and volume fraction of the γ′ phase.

Nano-sized γ′ precipitates with an average size of around 22 nm produced by air-cooling following the solution treatment should belong to the cutting mechanism, while the coarse γ′ precipitates with an average size of around 250–500 nm produced by slow cooling should belong to the Orowan by-pass mechanism. According to Equations (2) and (4), the precipitation strengthening is proportional to the faction and inversely proportional to the size of γ′. Hence, the coarse γ′ precipitates produced by slow cooling have a weak hindering effect on the dislocation motion, which reduced the precipitation strengthening effect of γ′, which is reflected in the decrease in the overall tensile strength. On the other hand, the ease regarding the passing of dislocation across the coarse γ′ resulted in an increase in plasticity. This is consistent with the previous research [41,42] that, regarding the coarse γ′, the dislocations are more prone to travel in groups within the same matrix channel, which decreases the stress required for precipitate shearing and thus results in decreased tensile strength and in turn makes the alloy more susceptible to be plasticly deformed.

According to the above results and discussion, the tensile strength and elongation of the alloy are quite different under varying solution temperatures and cooling rates following solution treatments. This is mainly attributed to the varying γ′ values obtained by solution treatments.

## 4. Conclusions

(1) During the sub-solvus solution treatment at 1040 °C, the primary coarse γ′ precipitates partially remained, while they were completely dissolved and the nano-sized secondary γ′ precipitates were precipitated during the super-solvus solution treatment above 1080 °C. The secondary γ′ precipitates grew slightly with the increase in the solution treatment temperature. This is reflected in the increased and then decreased tensile strength with the increase in the solution temperature from 1040 °C to 1120 °C.
(2) Solution time had a negligible influence on γ′ precipitation due to the complete dissolution of γ′ after the 1 h 1080 °C solution treatment. Increasing the solution treatment time caused a slight grain coarsening, and the solution time has a weak influence on the tensile strength of the forged Rene 41 superalloy.
(3) The cooling rate following the solution treatment plays a significant role in the γ′ size and morphology. The morphology of the γ′ precipitates changed gradually from spherical to concave cubic and octo-cubic with the decrease in the cooling rate after the solution treatment. Moreover, the strength was significantly decreased, while the inverse occurred regarding the change in ductility.

## Figures and Tables

**Table 1:** Chemical composition of Rene 41 superalloy (mass%).

Cr	Co	Mo	Al	Ti	B	C	Ni
19.58	11.35	10.40	1.4	3.1	0.007	0.09	Bal.

**Table 2:** Secondary γ′ content at different solution temperatures.

Solution Temp./°C	1040	1080	1120
ƒ_sec γ′_/%	26.1	26.2	26.7

**Table 3:** Secondary γ′ faction after solution treated and cooled at varying cooling rates following solution treatment.

Cooling Rate/(°C/h)	50	100	200	AC ≈ 10^4^
ƒ_γ′/_%	21.5	19.9	24.5	26.2

## Data Availability

The raw data supporting the conclusions of this article will be made available by the authors upon request.
